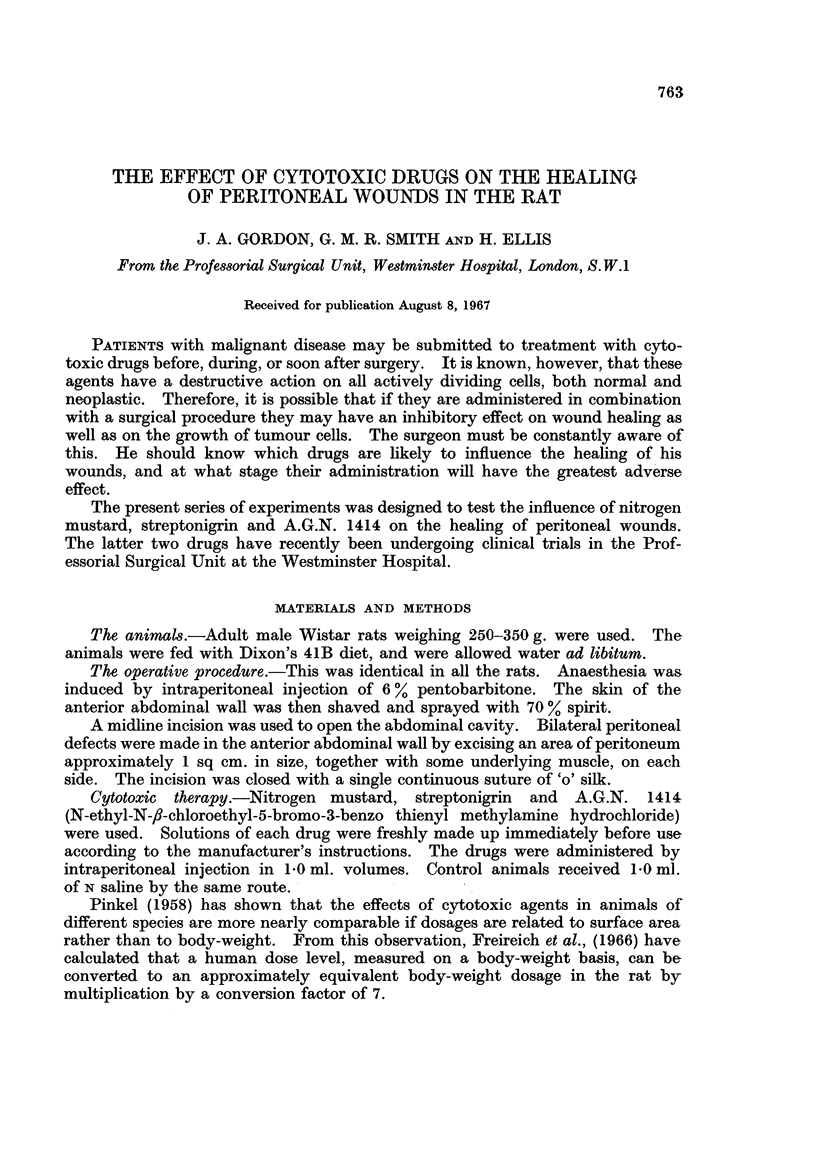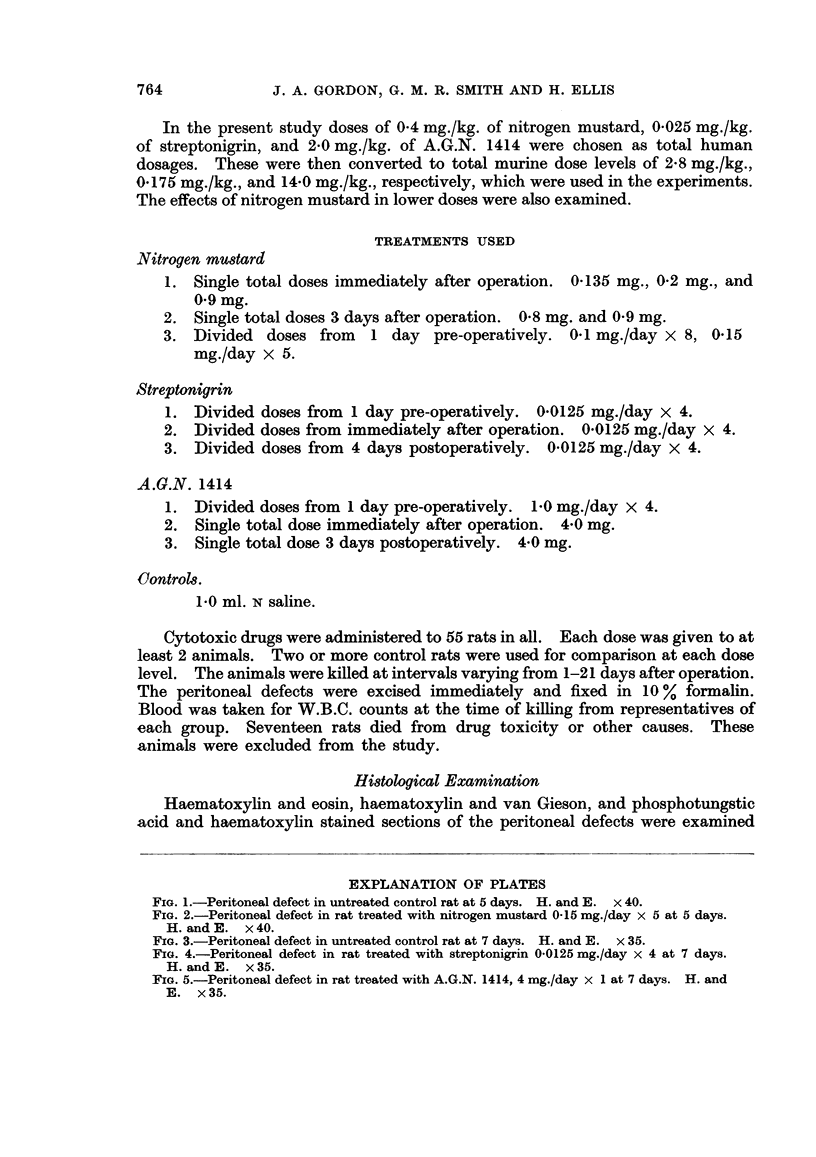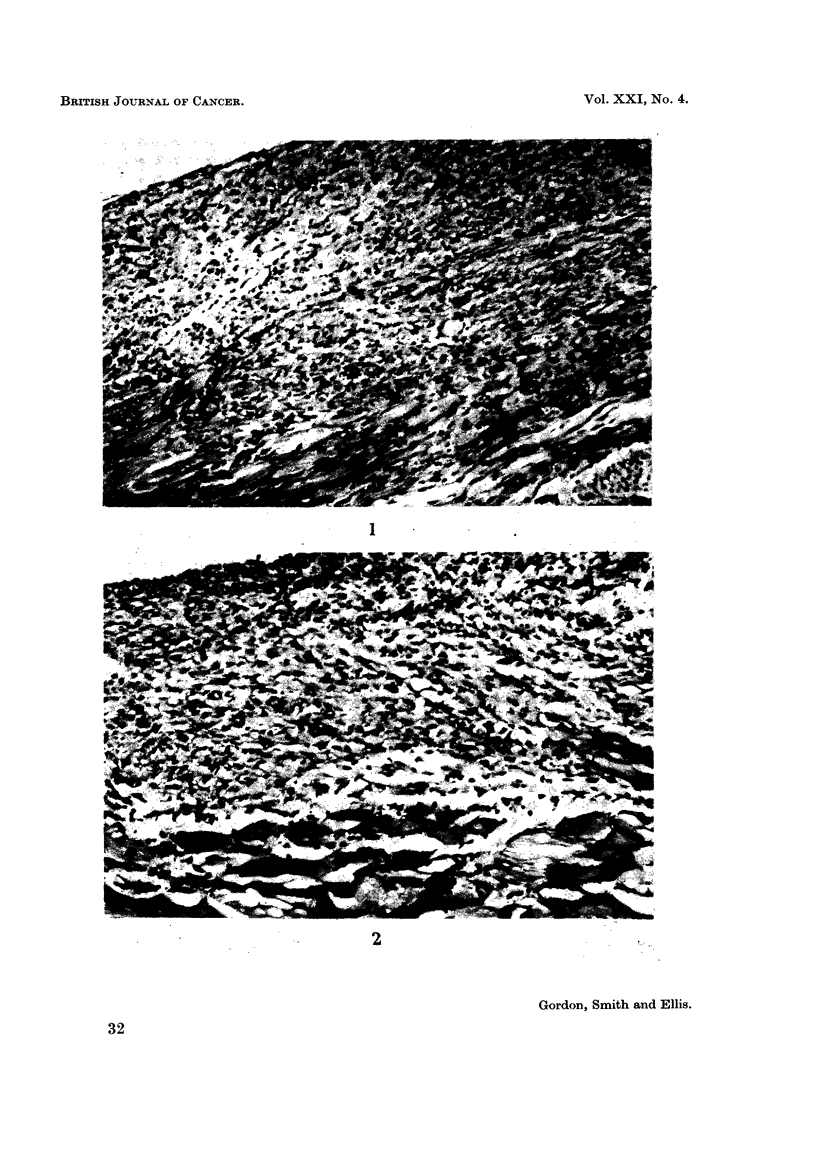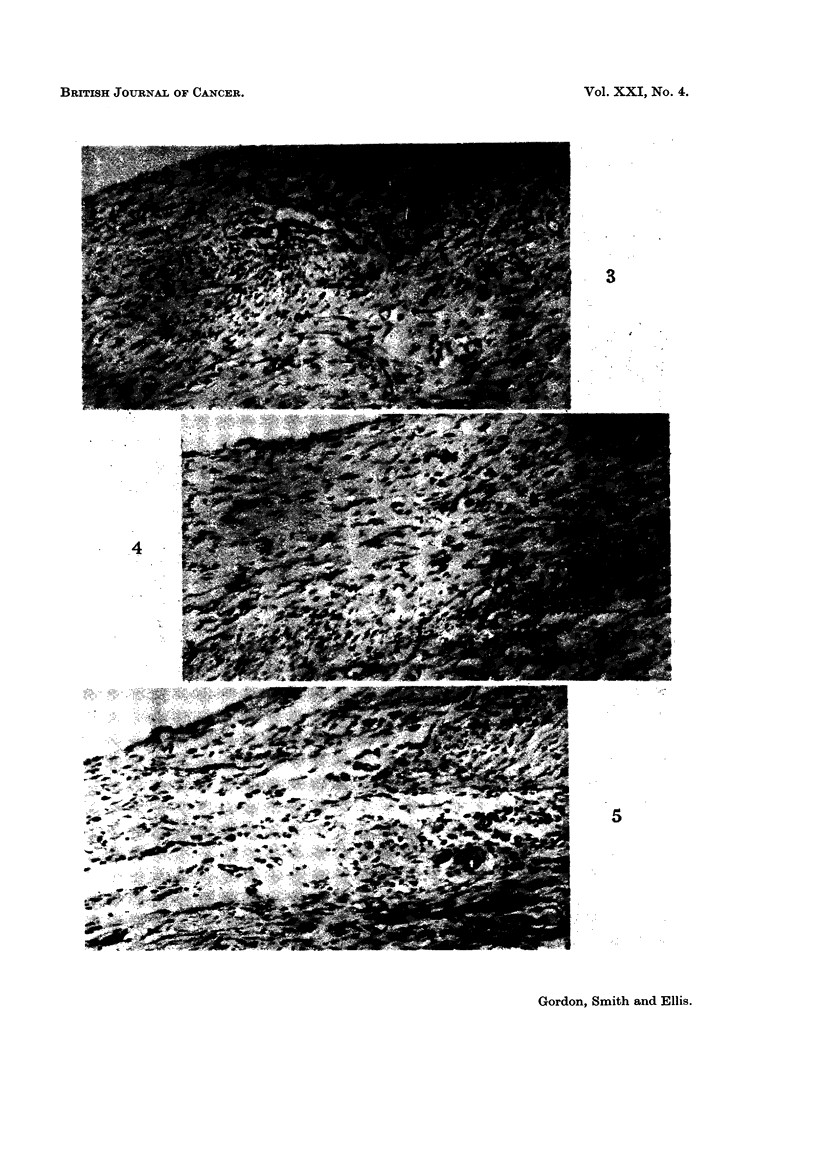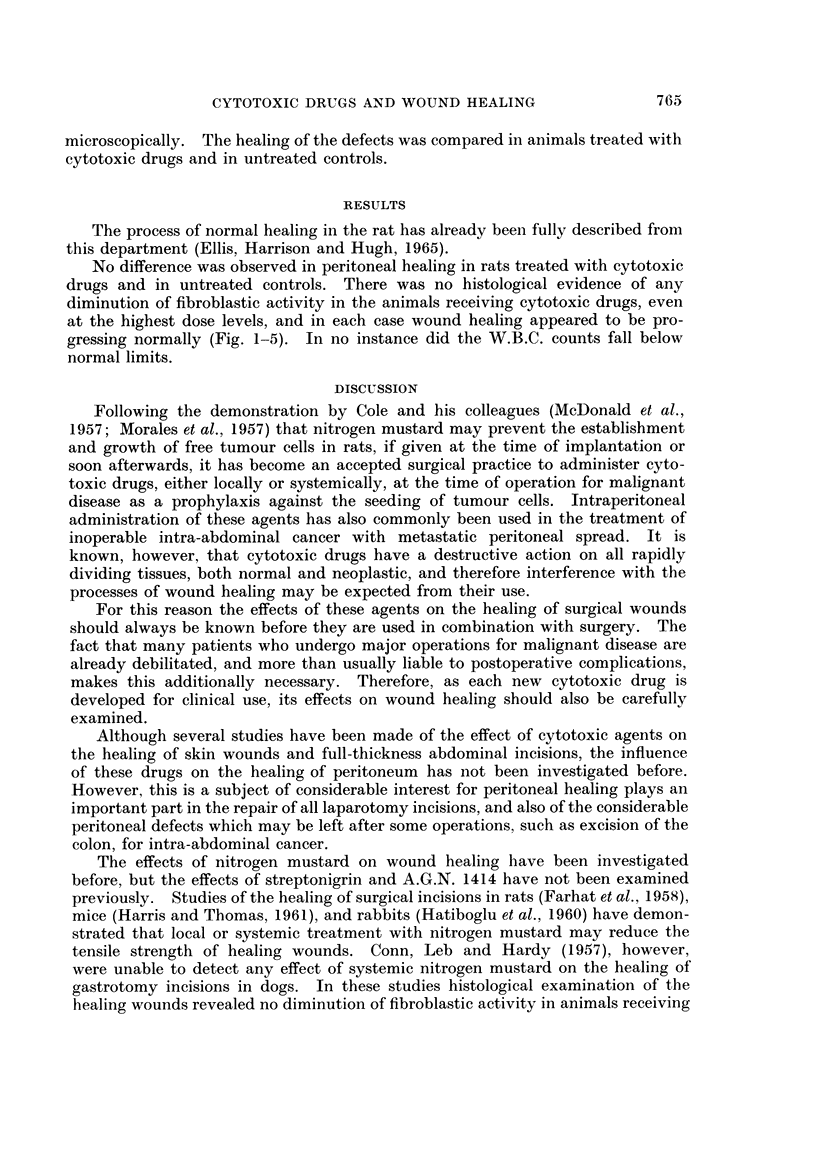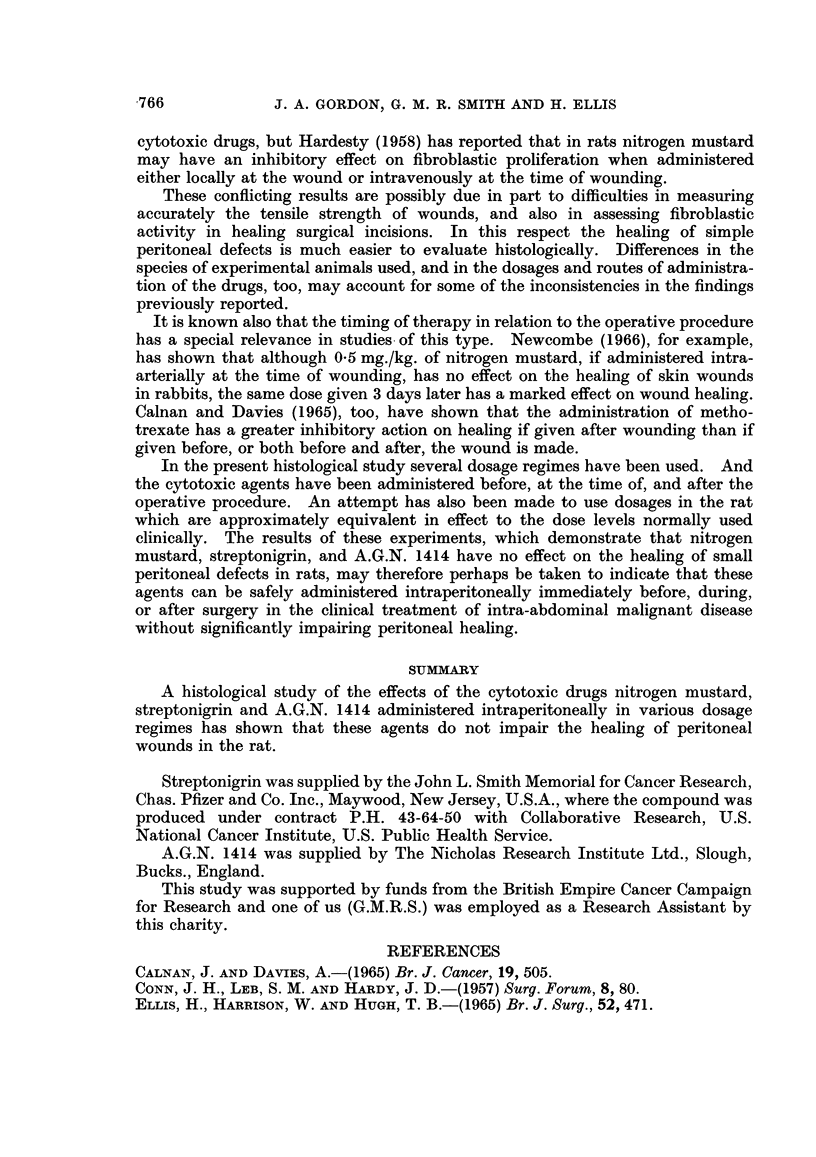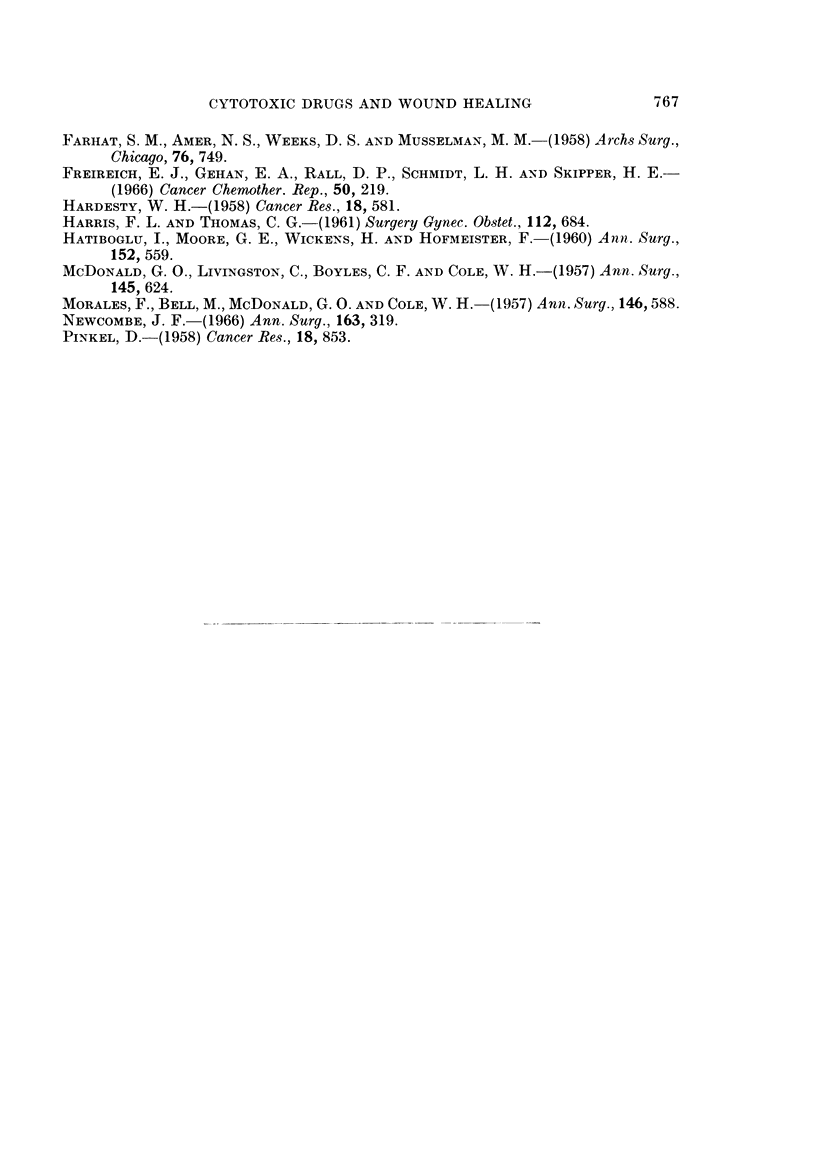# The effect of cytotoxic drugs on the healing of peritoneal wounds in the rat.

**DOI:** 10.1038/bjc.1967.89

**Published:** 1967-12

**Authors:** J. A. Gordon, G. M. Smith, H. Ellis

## Abstract

**Images:**


### THE EFFECT OF CYTOTOXIC DRUGS ON THE HEALING OF PERITONEAL WOUNDS IN THE RAT

J. A. GORDON, G. M. R. SMITH AND H. ELLIS

*From the Professorial Surgical Unit, Westminster Hospital, London, S.W.*1

Received for publication August 8, 1967

PATIENTS with malignant disease may be submitted to treatment with cytotoxic drugs before, during, or soon after surgery. It is known, however, that these agents have a destructive action on all actively dividing cells, both normal and neoplastic. Therefore, it is possible that if they are administered in combination with a surgical procedure they may have an inhibitory effect on wound healing as well as on the growth of tumour cells. The surgeon must be constantly aware of this. He should know which drugs are likely to influence the healing of his wounds, and at what stage their administration will have the greatest adverse effect.

The present series of experiments was designed to test the influence of nitrogen mustard, streptonigrin and A.G.N. 1414 on the healing of peritoneal wounds. The latter two drugs have recently been undergoing clinical trials in the Professorial Surgical Unit at the Westminster Hospital.

### MATERIALS AND METHODS

*The animals.*—Adult male Wistar rats weighing 250–350 g. were used. The animals were fed with Dixon's 41B diet, and were allowed water *ad libitum.*

*The operative procedure.*—This was identical in all the rats. Anaesthesia was induced by intraperitoneal injection of 6% pentobarbitone. The skin of the anterior abdominal wall was then shaved and sprayed with 70% spirit.

A midline incision was used to open the abdominal cavity. Bilateral peritoneal defects were made in the anterior abdominal wall by excising an area of peritoneum approximately 1 sq cm. in size, together with some underlying muscle, on each side. The incision was closed with a single continuous suture of 'o' silk.

*Cytotoxic therapy.*—Nitrogen mustard, streptonigrin and A.G.N. 1414 (N-ethyl-N-$\beta$-chloroethyl-5-bromo-3-benzo thienyl methylamine hydrochloride) were used. Solutions of each drug were freshly made up immediately before use according to the manufacturer's instructions. The drugs were administered by intraperitoneal injection in 1·0 ml. volumes. Control animals received 1·0 ml. of N saline by the same route.

Pinkel (1958) has shown that the effects of cytotoxic agents in animals of different species are more nearly comparable if dosages are related to surface area rather than to body-weight. From this observation, Freireich *et al.*, (1966) have calculated that a human dose level, measured on a body-weight basis, can be converted to an approximately equivalent body-weight dosage in the rat by multiplication by a conversion factor of 7.

In the present study doses of 0·4 mg./kg. of nitrogen mustard, 0·025 mg./kg. of streptonigrin, and 2·0 mg./kg. of A.G.N. 1414 were chosen as total human dosages. These were then converted to total murine dose levels of 2·8 mg./kg., 0·175 mg./kg., and 14·0 mg./kg., respectively, which were used in the experiments. The effects of nitrogen mustard in lower doses were also examined.

### TREATMENTS USED

*Nitrogen mustard*

1. Single total doses immediately after operation. 0·135 mg., 0·2 mg., and 0·9 mg.
2. Single total doses 3 days after operation. 0·8 mg. and 0·9 mg.
3. Divided doses from 1 day pre-operatively. 0·1 mg./day × 8, 0·15 mg./day × 5.

*Streptonigrin*

1. Divided doses from 1 day pre-operatively. 0·0125 mg./day × 4.
2. Divided doses from immediately after operation. 0·0125 mg./day × 4.
3. Divided doses from 4 days postoperatively. 0·0125 mg./day × 4.

*A.G.N.* 1414

1. Divided doses from 1 day pre-operatively. 1·0 mg./day × 4.
2. Single total dose immediately after operation. 4·0 mg.
3. Single total dose 3 days postoperatively. 4·0 mg.

*Controls.*

1·0 ml. N saline.

Cytotoxic drugs were administered to 55 rats in all. Each dose was given to at least 2 animals. Two or more control rats were used for comparison at each dose level. The animals were killed at intervals varying from 1–21 days after operation. The peritoneal defects were excised immediately and fixed in 10% formalin. Blood was taken for W.B.C. counts at the time of killing from representatives of each group. Seventeen rats died from drug toxicity or other causes. These animals were excluded from the study.

### *Histological Examination*

Haematoxylin and eosin, haematoxylin and van Gieson, and phosphotungstic acid and haematoxylin stained sections of the peritoneal defects were examined

---

EXPLANATION OF PLATES

FIG. 1.—Peritoneal defect in untreated control rat at 5 days. H. and E. ×40.
FIG. 2.—Peritoneal defect in rat treated with nitrogen mustard 0·15 mg./day × 5 at 5 days. H. and E. ×40.
FIG. 3.—Peritoneal defect in untreated control rat at 7 days. H. and E. ×35.
FIG. 4.—Peritoneal defect in rat treated with streptonigrin 0·0125 mg./day × 4 at 7 days. H. and E. ×35.
FIG. 5.—Peritoneal defect in rat treated with A.G.N. 1414, 4 mg./day × 1 at 7 days. H. and E. ×35.

1

2

Gordon, Smith and Ellis.

3

4

5

Gordon, Smith and Ellis.

microscopically. The healing of the defects was compared in animals treated with cytotoxic drugs and in untreated controls.

### RESULTS

The process of normal healing in the rat has already been fully described from this department (Ellis, Harrison and Hugh, 1965).

No difference was observed in peritoneal healing in rats treated with cytotoxic drugs and in untreated controls. There was no histological evidence of any diminution of fibroblastic activity in the animals receiving cytotoxic drugs, even at the highest dose levels, and in each case wound healing appeared to be progressing normally (Fig. 1–5). In no instance did the W.B.C. counts fall below normal limits.

### DISCUSSION

Following the demonstration by Cole and his colleagues (McDonald *et al.*, 1957; Morales *et al.*, 1957) that nitrogen mustard may prevent the establishment and growth of free tumour cells in rats, if given at the time of implantation or soon afterwards, it has become an accepted surgical practice to administer cytotoxic drugs, either locally or systemically, at the time of operation for malignant disease as a prophylaxis against the seeding of tumour cells. Intraperitoneal administration of these agents has also commonly been used in the treatment of inoperable intra-abdominal cancer with metastatic peritoneal spread. It is known, however, that cytotoxic drugs have a destructive action on all rapidly dividing tissues, both normal and neoplastic, and therefore interference with the processes of wound healing may be expected from their use.

For this reason the effects of these agents on the healing of surgical wounds should always be known before they are used in combination with surgery. The fact that many patients who undergo major operations for malignant disease are already debilitated, and more than usually liable to postoperative complications, makes this additionally necessary. Therefore, as each new cytotoxic drug is developed for clinical use, its effects on wound healing should also be carefully examined.

Although several studies have been made of the effect of cytotoxic agents on the healing of skin wounds and full-thickness abdominal incisions, the influence of these drugs on the healing of peritoneum has not been investigated before. However, this is a subject of considerable interest for peritoneal healing plays an important part in the repair of all laparotomy incisions, and also of the considerable peritoneal defects which may be left after some operations, such as excision of the colon, for intra-abdominal cancer.

The effects of nitrogen mustard on wound healing have been investigated before, but the effects of streptonigrin and A.G.N. 1414 have not been examined previously. Studies of the healing of surgical incisions in rats (Farhat *et al.*, 1958), mice (Harris and Thomas, 1961), and rabbits (Hatiboglu *et al.*, 1960) have demonstrated that local or systemic treatment with nitrogen mustard may reduce the tensile strength of healing wounds. Conn, Leb and Hardy (1957), however, were unable to detect any effect of systemic nitrogen mustard on the healing of gastrotomy incisions in dogs. In these studies histological examination of the healing wounds revealed no diminution of fibroblastic activity in animals receiving cytotoxic drugs, but Hardesty (1958) has reported that in rats nitrogen mustard may have an inhibitory effect on fibroblastic proliferation when administered either locally at the wound or intravenously at the time of wounding.

These conflicting results are possibly due in part to difficulties in measuring accurately the tensile strength of wounds, and also in assessing fibroblastic activity in healing surgical incisions. In this respect the healing of simple peritoneal defects is much easier to evaluate histologically. Differences in the species of experimental animals used, and in the dosages and routes of administration of the drugs, too, may account for some of the inconsistencies in the findings previously reported.

It is known also that the timing of therapy in relation to the operative procedure has a special relevance in studies of this type. Newcombe (1966), for example, has shown that although 0·5 mg./kg. of nitrogen mustard, if administered intra-arterially at the time of wounding, has no effect on the healing of skin wounds in rabbits, the same dose given 3 days later has a marked effect on wound healing. Calnan and Davies (1965), too, have shown that the administration of methotrexate has a greater inhibitory action on healing if given after wounding than if given before, or both before and after, the wound is made.

In the present histological study several dosage regimes have been used. And the cytotoxic agents have been administered before, at the time of, and after the operative procedure. An attempt has also been made to use dosages in the rat which are approximately equivalent in effect to the dose levels normally used clinically. The results of these experiments, which demonstrate that nitrogen mustard, streptonigrin, and A.G.N. 1414 have no effect on the healing of small peritoneal defects in rats, may therefore perhaps be taken to indicate that these agents can be safely administered intraperitoneally immediately before, during, or after surgery in the clinical treatment of intra-abdominal malignant disease without significantly impairing peritoneal healing.

### SUMMARY

A histological study of the effects of the cytotoxic drugs nitrogen mustard, streptonigrin and A.G.N. 1414 administered intraperitoneally in various dosage regimes has shown that these agents do not impair the healing of peritoneal wounds in the rat.

Streptonigrin was supplied by the John L. Smith Memorial for Cancer Research, Chas. Pfizer and Co. Inc., Maywood, New Jersey, U.S.A., where the compound was produced under contract P.H. 43-64-50 with Collaborative Research, U.S. National Cancer Institute, U.S. Public Health Service.

A.G.N. 1414 was supplied by The Nicholas Research Institute Ltd., Slough, Bucks., England.

This study was supported by funds from the British Empire Cancer Campaign for Research and one of us (G.M.R.S.) was employed as a Research Assistant by this charity.

### REFERENCES

Calnan, J. and Davies, A.—(1965) *Br. J. Cancer*, **19**, 505.
Conn, J. H., Leb, S. M. and Hardy, J. D.—(1957) *Surg. Forum*, **8**, 80.
Ellis, H., Harrison, W. and Hugh, T. B.—(1965) *Br. J. Surg.*, **52**, 471.

FARHAT, S. M., AMER, N. S., WEEKS, D. S. AND MUSSELMAN, M. M.—(1958) *Archs Surg., Chicago*, **76,** 749.
FREIREICH, E. J., GEHAN, E. A., RALL, D. P., SCHMIDT, L. H. AND SKIPPER, H. E.—(1966) *Cancer Chemother. Rep.*, **50,** 219.
HARDESTY, W. H.—(1958) *Cancer Res.*, **18,** 581.
HARRIS, F. L. AND THOMAS, C. G.—(1961) *Surgery Gynec. Obstet.*, **112,** 684.
HATIBOGLU, I., MOORE, G. E., WICKENS, H. AND HOFMEISTER, F.—(1960) *Ann. Surg.*, **152,** 559.
McDONALD, G. O., LIVINGSTON, C., BOYLES, C. F. AND COLE, W. H.—(1957) *Ann. Surg.*, **145,** 624.
MORALES, F., BELL, M., McDONALD, G. O. AND COLE, W. H.—(1957) *Ann. Surg.*, **146,** 588.
NEWCOMBE, J. F.—(1966) *Ann. Surg.*, **163,** 319.
PINKEL, D.—(1958) *Cancer Res.*, **18,** 853.